# Supplementary material for: Tagging active neurons by soma-targeted Cal-Light
Source: Nat Commun. 2022 Dec 12;13:7692. doi: 10.1038/s41467-022-35406-y (PMC9744738; doi:10.1038/s41467-022-35406-y)
Supplement: Supplementary file 1 — Supplementary Information [file 41467_2022_35406_MOESM1_ESM.pdf]

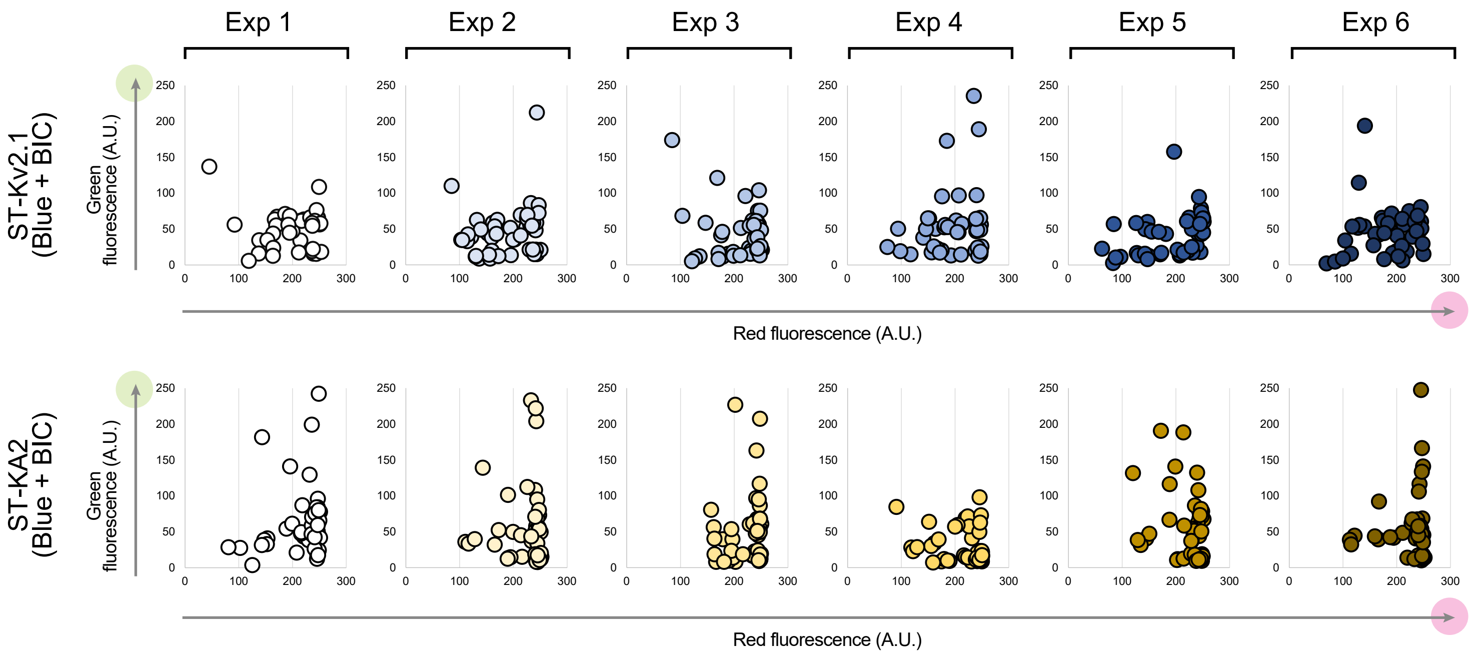

Supplementary Fig. 1. Fluorescence (Red and Green) distribution by each experiment. To assess the degree of variation caused by virus injection on different sets of experiments, fluorescence signals were plotted by independent experiments. Both ST-Kv2.1 and ST-KA2 were displayed in the presence of blue light and bicuculline.

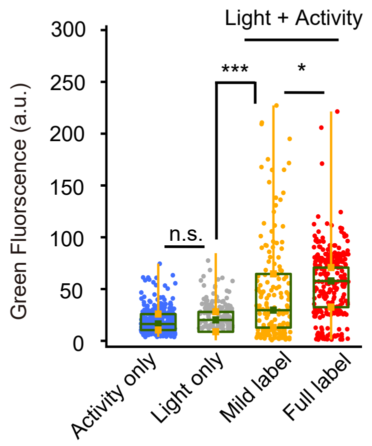

Supplementary Fig. 2. Analysis of green signals only from Fig. 2f. To exclude the possibility of skewed G/R by thresholding red fluorescence signals, only green signal (reporter gene) was analyzed. Note that the significant increase of green signals was affected by light and activity. (Activity only: 19.41 ± 0.654, n = 223 cells; Light only: 20.26 ± 0.826, n = 210 cells, $P = 0.98$ compared to Activity only; Mild label: 47.8 ± 2.352, n = 234 cells, $P = 5.38 \times 10^{-20}$ compared to Light only; Full label: 54.1 ± 1.287 n = 238 cells, $P = 0.00169$ compared to Mild label). Box plots show the median, 25th and 75th percentiles and whiskers show min and max. Statistics is analyzed by one-way ANOVA ($P < 0.0001$). Asterisks (*$P < 0.05$ and ***$P < 0.005$) indicate Bonferroni post-hoc significance.

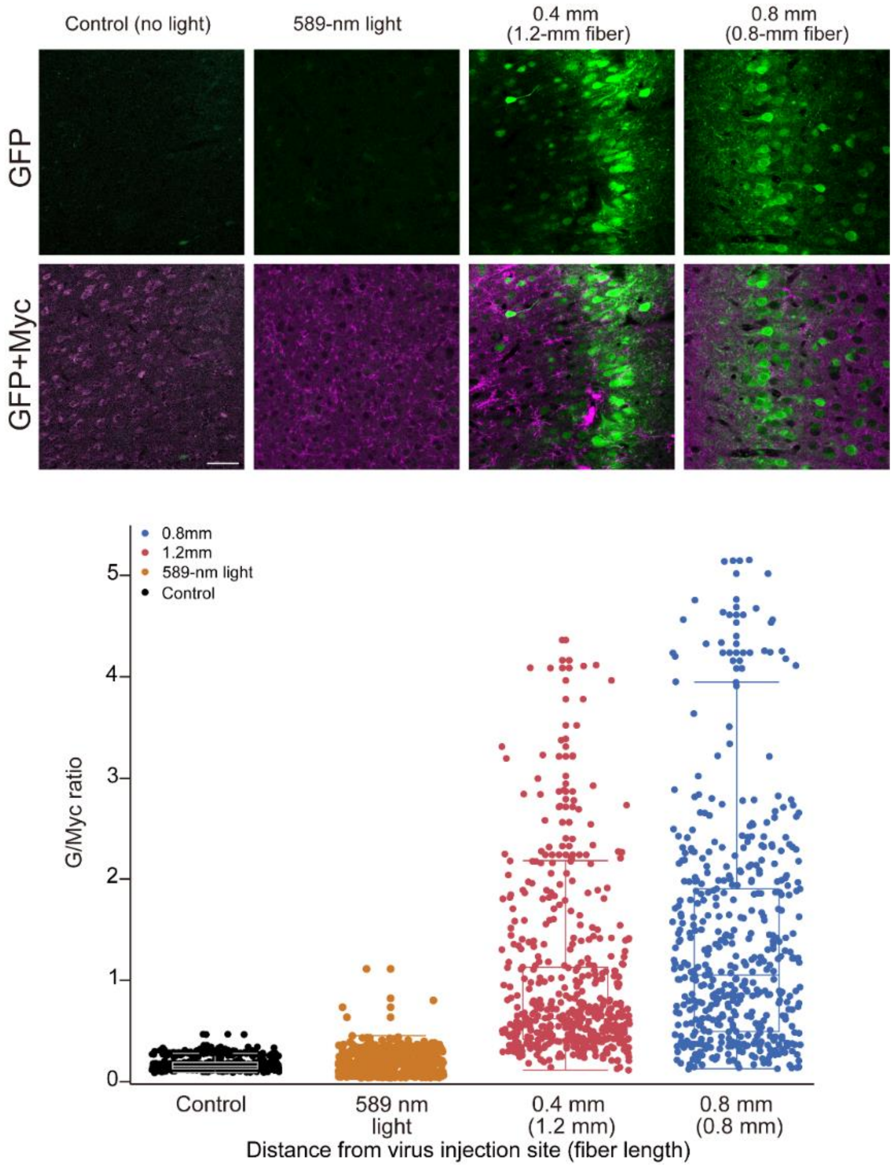

Supplementary Fig. 3. The level of gene expression in the absence of blue light or by 589 nm light. When blue light was not delivered or 589 nm light (2 sec ON/1 sec OFF, 30 min in the open field chamber) was used for labeling, no EGFP reporter was expressed. When the tip of optic fiber was located 0.4 or 0.8 mm above the virus injection site, the labeling efficiencies were similar. Myc epitope was detected by antibody staining (Control: 0.1667 ± 0.00301, n = 453 cells from 2 mice; 589 nm light: 0.1768 ± 0.00545, n = 508 cells from 3 mice; 0.4 mm: 0.7896 ± 0.0316, n = 344 cells from 3 mice; 0.8 mm: 1.1146 ± 0.0416, n = 338 cells from 3 mice). The top and bottom of the box indicate 25th and 75th percentile respectively, horizontal line in the box is the median, and the upper and lower whiskers mean the minimum and maximum values. Scale, 50 μm.

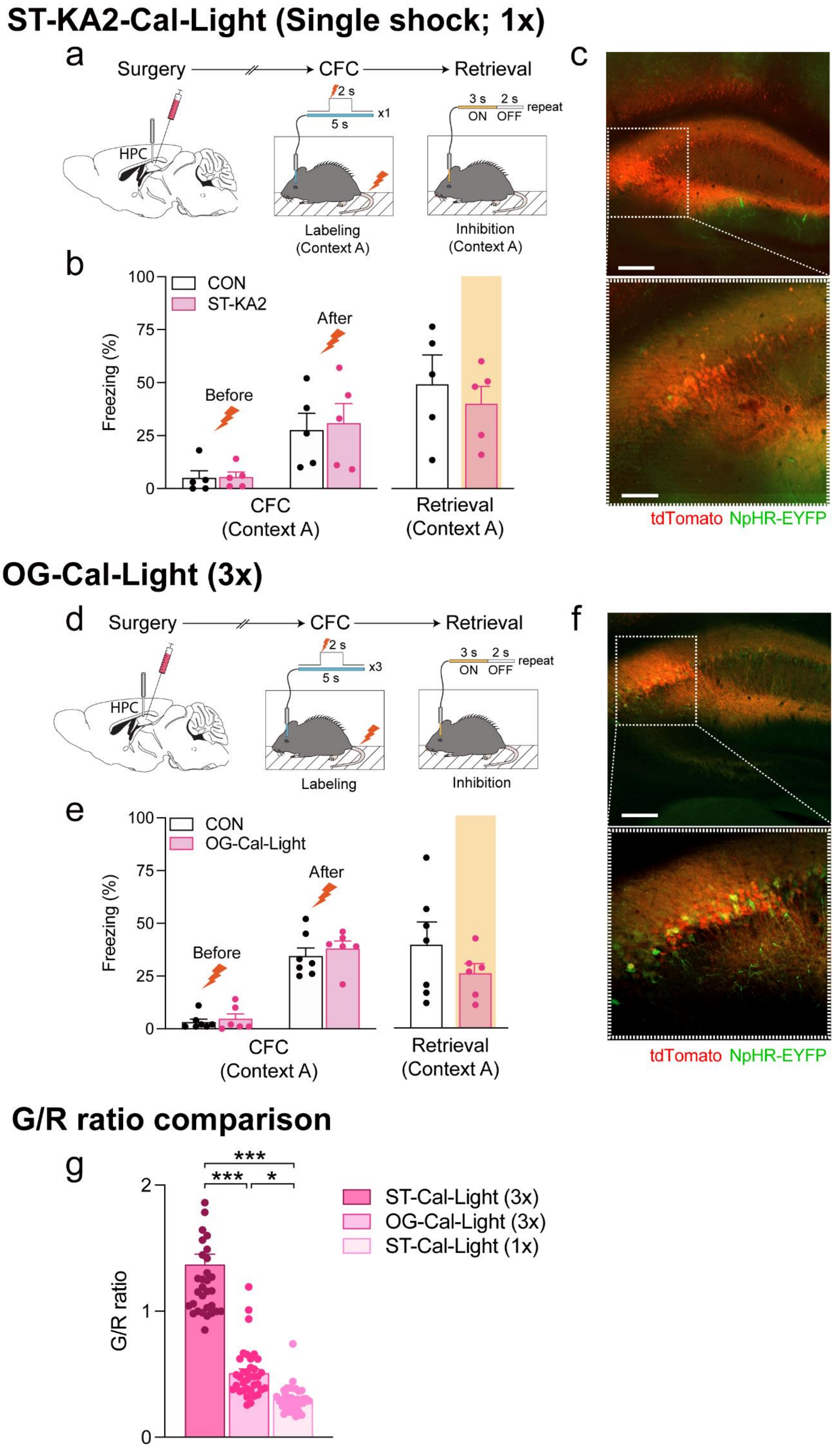

ST-KA2-Cal-Light (Single shock; 1x)
a
Surgery
CFC
Retrieval
HPC
2 s
5 s
x1
3 s
2 s
ON
OFF
repeat
Labeling
(Context A)
Inhibition
(Context A)
b
CON
ST-KA2
Freezing (%)
100
75
50
25
0
Before
After
CFC
(Context A)
Retrieval
(Context A)
c
tdTomato NpHR-EYFP
OG-Cal-Light (3x)
d
Surgery
CFC
Retrieval
HPC
2 s
5 s
x3
3 s
2 s
ON
OFF
repeat
Labeling
Inhibition
e
CON
OG-Cal-Light
Freezing (%)
100
75
50
25
0
Before
After
CFC
(Context A)
Retrieval
(Context A)
f
tdTomato NpHR-EYFP
G/R ratio comparison
g
***
***
*
2
1
0
G/R ratio
ST-Cal-Light (3x)
OG-Cal-Light (3x)
ST-Cal-Light (1x)

Supplementary Fig. 4. Comparison of ST-KA2-Cal-Light and OG-Cal-Light. a. Overall scheme of experimental procedure. Neurons activated by single foot shock was labeled by ST-KA2-Cal-Light. **b.** Single foot shock was sufficient to cause animals to freeze in the conditioned context. Suppression of labeled neurons by yellow light (589 nm) did not prevent freezing behavior (Control: from n = 5 independent mice; ST-KA2: from 5 independent mice). Data are presented with mean ± S.E.M. **c.** Post-hoc imaging revealed weak labeling of active neurons by delivering blue light only for 5 sec. Scale, 50 and 10 μm each. **d.** Experimental time line of labeling and retrieval of active neurons. Viruses expressing OG-Cal-Light was injected in the hippocampus bilaterally. Labeling was made with three times of blue light concomitantly with foot shocks. **e.** The magnitude of freezing behavior before and after fear conditioning, and in the presence of yellow light. Labeling was not strong enough to show behavioral changes when the OG-Cal-Light was used. (Control: from n = 7 independent mice; OG-Cal-Light: from 6 independent mice). Data are presented with mean ± S.E.M. **f.** Representative images of NpHR-EYFP signals. Scale, 50 μm. **g.** Comparison of labeling efficiency by ST-Cal-Light and OG-Cal-Light. (ST-Cal-Light (3x): n = 34 independent cells, OG-Cal-Light: n = 36 independent cells, ST-Cal-Light (1x): n = 36 independent cells). Data are presented with mean ± S.E.M. For all graphs, * and *** indicate $P < 0.05$ and $P < 0.005$, respectively.

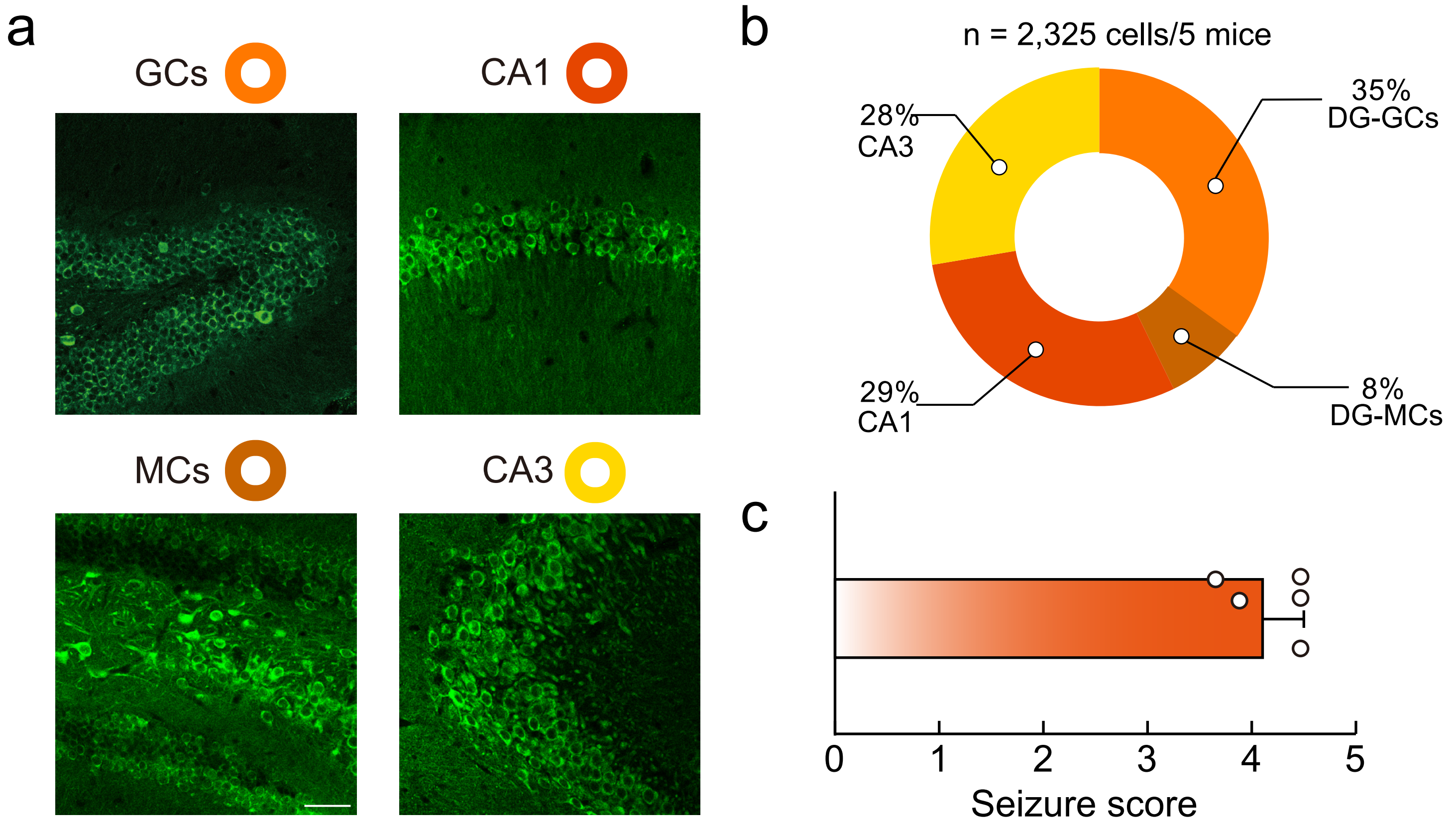

Supplementary Fig. 5. Hippocampal neuron labeling during the lever pressing behavior. a. Images of EGFP expression in the various regions of hippocampus. Blue light was illuminated during the lever pressing training to match the same number of blue light repeats and the total duration of light exposure that was given during the seizure experiment. Scale bar, 50 µm. b. Quantification of labeled neurons in CA1, CA3, dentate gyrus granule cells, and mossy cells (n = 2325 cells, 5 mice). c. Seizure score in the absence of yellow light after KA injection. Note that inhibiting lever pressing relevant neurons did not reduce epileptic seizure symptoms (Seizure score = 4.1 ± 0.113, 5 mice). Data are presented as mean value ± SEM.

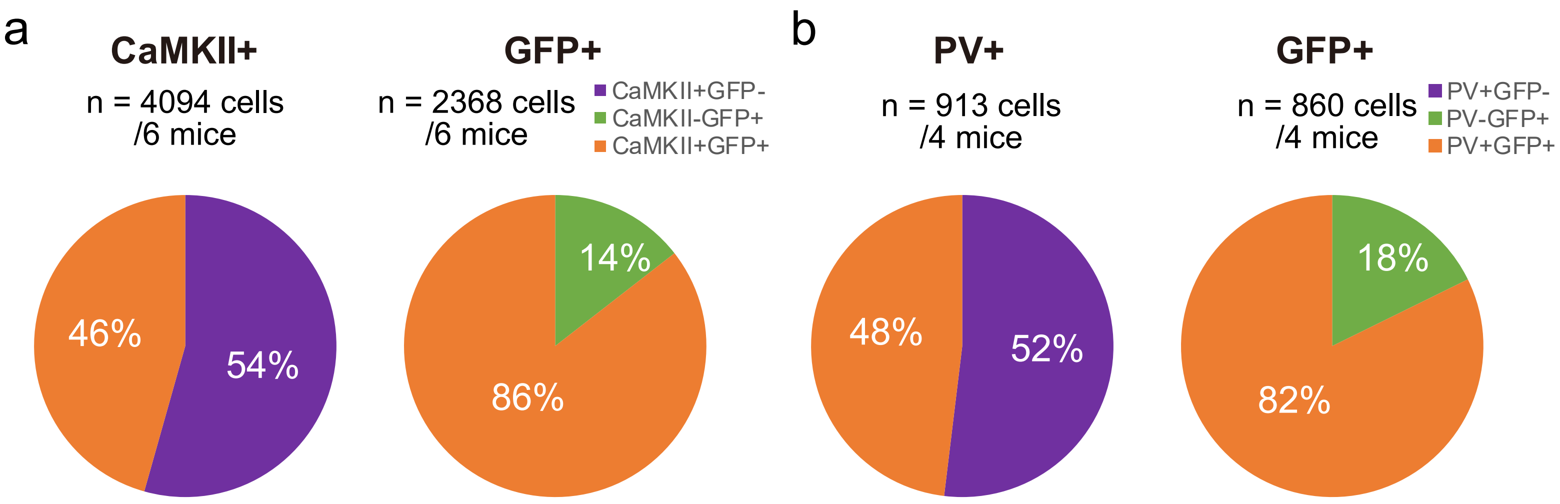

Supplementary Fig. 6. Colocalization of CaMKII- and GFP-positive neurons, and PV- and GFP-positive neurons. a,b. Analysis of the percentage of EGFP+ neurons out of CaMKII+ neurons or PV+ neurons. We also analyzed the percentage of EGFP+ neurons colocalizing with PV or CaMKII.
