# Supplementary material for: Tagging active neurons by soma-targeted Cal-Light
Source: Nat Commun. 2022 Dec 12;13:7692. doi: 10.1038/s41467-022-35406-y (PMC9744738; doi:10.1038/s41467-022-35406-y)
Supplement: Supplementary file 2 — Reporting Summary [file 41467_2022_35406_MOESM2_ESM.pdf]

nature portfolio

Corresponding author(s): Hyung-Bae Kwon

Last updated by author(s): Nov 29, 2022

Reporting Summary

Nature Portfolio wishes to improve the reproducibility of the work that we publish. This form provides structure for consistency and transparency in reporting. For further information on Nature Portfolio policies, see our Editorial Policies and the Editorial Policy Checklist.

Statistics

For all statistical analyses, confirm that the following items are present in the figure legend, table legend, main text, or Methods section.

n/a	Confirmed	
[ ]	[x]	The exact sample size (*n*) for each experimental group/condition, given as a discrete number and unit of measurement
[ ]	[x]	A statement on whether measurements were taken from distinct samples or whether the same sample was measured repeatedly
[ ]	[x]	The statistical test(s) used AND whether they are one- or two-sided<br>*Only common tests should be described solely by name; describe more complex techniques in the Methods section.*
[x]	[ ]	A description of all covariates tested
[ ]	[x]	A description of any assumptions or corrections, such as tests of normality and adjustment for multiple comparisons
[ ]	[x]	A full description of the statistical parameters including central tendency (e.g. means) or other basic estimates (e.g. regression coefficient) AND variation (e.g. standard deviation) or associated estimates of uncertainty (e.g. confidence intervals)
[ ]	[x]	For null hypothesis testing, the test statistic (e.g. *F*, *t*, *r*) with confidence intervals, effect sizes, degrees of freedom and *P* value noted<br>*Give P values as exact values whenever suitable.*
[x]	[ ]	For Bayesian analysis, information on the choice of priors and Markov chain Monte Carlo settings
[x]	[ ]	For hierarchical and complex designs, identification of the appropriate level for tests and full reporting of outcomes
[ ]	[x]	Estimates of effect sizes (e.g. Cohen's *d*, Pearson's *r*), indicating how they were calculated

Our web collection on statistics for biologists contains articles on many of the points above.

Software and code

Policy information about availability of computer code

Data collection	ZEN 2012, IgorPro6.10A, MED-PC IV
Data analysis	IgorPro6.10A, JMP Pro, OriginPro 2020b

For manuscripts utilizing custom algorithms or software that are central to the research but not yet described in published literature, software must be made available to editors and reviewers. We strongly encourage code deposition in a community repository (e.g. GitHub). See the Nature Portfolio guidelines for submitting code & software for further information.

Data

Policy information about availability of data

All manuscripts must include a data availability statement. This statement should provide the following information, where applicable:

- Accession codes, unique identifiers, or web links for publicly available datasets
- A description of any restrictions on data availability
- For clinical datasets or third party data, please ensure that the statement adheres to our policy

All plasmids used in this study were deposited and available in Addgene (ID: 171615 ~ 171622). The conditional ST-Cal-Light KI mice are available from the corresponding author upon request. Source data are provided with this paper. The data that support the findings of this study are available from the corresponding author upon reasonable request.

Human research participants

Policy information about studies involving human research participants and Sex and Gender in Research.

Reporting on sex and gender	Not applicable
Population characteristics	Not applicable
Recruitment	Not applicable
Ethics oversight	Not applicable

Note that full information on the approval of the study protocol must also be provided in the manuscript.

Field-specific reporting

Please select the one below that is the best fit for your research. If you are not sure, read the appropriate sections before making your selection.

☒ Life sciences ☐ Behavioural & social sciences ☐ Ecological, evolutionary & environmental sciences

For a reference copy of the document with all sections, see nature.com/documents/nr-reporting-summary-flat.pdf

Life sciences study design

All studies must disclose on these points even when the disclosure is negative.

Sample size	No statistical methods were used to pre-determine sample sizes but the sample sizes used in our study, such as the numbers of neurons or animals, are about the same or similar to the previous publication (Lee et al., 2017).
Data exclusions	We excluded the cells that did not have tdTomato signals (transfection marker).<br>Mice that did not learn lever-pressing behaviors during the first phase of training (Day 1-4).
Replication	To ensure that our experimental findings can be reliably replicated, each experiment was repeated at least twice by multiple experimenters. For imaging and behavioral experiments where carried out in individual cohorts that were tested on separate occasions. All attempts of replicating the data were successful.
Randomization	All subjects were randomly allocated to the different experimental conditions.
Blinding	All cell counting and behavioral experiments were performed by an experimenter blind to the experimental condition only except for the ST-KA2-Cal cKI mice experiment (Fig. 5). Blinding to the experimental condition was not possible in this newly generated mice study due to the researchers has to know the genotype since the cKI mice were generated for the 1st time in the world and we have to very carefully identify the genotypes for the future purpose and possible distributions to the community.

Reporting for specific materials, systems and methods

We require information from authors about some types of materials, experimental systems and methods used in many studies. Here, indicate whether each material, system or method listed is relevant to your study. If you are not sure if a list item applies to your research, read the appropriate section before selecting a response.

Materials & experimental systems

n/a	Involved in the study	
☐	☒	Antibodies
☐	☒	Eukaryotic cell lines
☒	☐	Palaeontology and archaeology
☐	☒	Animals and other organisms
☒	☐	Clinical data
☒	☐	Dual use research of concern

Methods

n/a	Involved in the study	
☒	☐	ChIP-seq
☒	☐	Flow cytometry
☒	☐	MRI-based neuroimaging

Antibodies

Antibodies used	The primary antibodies used were: anti-PV (1:500, Swant, PVG-213) or anti-CaMKII (1:500, Abcam, #ab22609).<br>The primary antibodies used for myc tag staining: the mixture of RFP antibody pre-absorbed (1:1000 in blocking reagent, Rockland

	antibodies & assays, 600-401-379) and anti-Myc tag antibody (1:1000, Abcam, #ab32) were used. The following secondary antibodies were used: Goat anti-Mouse IgG Alexa flour 633 (Thermo Fisher Scientific, #A21052), Goat anti-Rat IgG Alexa flour 647 (Thermo Fisher Scientific, #A21247), Alexa Fluor 488 Anti-Mouse (1:500, Donkey, Jackson ImmunoResearch, 715-545-150), and Cy3 Anti-Rabbit IgG (1:500, Donkey, Jackson ImmunoResearch, 711-165-152); mounted with DAPI Fluoromount-G (Southern BioTech, 0100-01).
Validation	All antibodies were validated by the companies we obtained them from prior to purchase. Additionally, all antibodies were verified many times by multiple lab members in house, and in all cases have been verified in peer-reviewed publications (Lee et al., 2017a; Lee et al., 2017b).

Eukaryotic cell lines

Policy information about cell lines and Sex and Gender in Research

Cell line source(s)	Not applicable. No cell lines were used.
Authentication	None of the cell lines have been authenticated
Mycoplasma contamination	Not applicable
Commonly misidentified lines (See ICLAC register)	No commonly misidentified cell lines were used in this study.

Animals and other research organisms

Policy information about studies involving animals; ARRIVE guidelines recommended for reporting animal research, and Sex and Gender in Research

Laboratory animals	The following mouse lines were used: EMX1-Cre (Stock No: 005628), and PV-Cre (Stock No: 017320). In addition, we used C57BL/6J strain mice (The Jackson Laboratory).<br>Homozygous ST-Cal-Light cKI mice were bred to homozygous PV-Cre, and EMX-Cre mice to produce Cal-Het::EMX-Cre, Cal-Het::PV-Cre mice. Both male and female mice 6–12 weeks of age were used for all experiments.<br>All mice for behaviour tests were individually housed in a 12-h dark-light reverse cycle and experiments were performed during the dark cycle period. Mice were maintained in conventional housing with free access to food ad libitum when not being tested.<br>Primary dissociated neuron cultures were obtained from embryonic day 18-19 Hippocampus of CD IGS rats.
Wild animals	No wild animals were used in this study.
Reporting on sex	Both male and female mice 6–12 weeks of age were used for all experiments (see above Laboratory animals questionaire)
Field-collected samples	No field-collected samples were used in this study.
Ethics oversight	All experimental procedures and protocols were conducted with the approval of the Max Planck Florida Institute for Neuroscience (MPFI) Institutional Animal Care and Use Committee (IACUC), Johns Hopkins University IACUC and National Institutes of Health (NIH) guidelines.

Note that full information on the approval of the study protocol must also be provided in the manuscript.
